# Optimization of the Production of Secondary Metabolites from Furanocoumarin and Furoquinoline Alkaloid Groups in In Vitro *Ruta corsica* Cultures Grown in Temporary Immersion Bioreactors

**DOI:** 10.3390/molecules29225261

**Published:** 2024-11-07

**Authors:** Agnieszka Szewczyk, Monika Trepa, Dominika Zych

**Affiliations:** 1 Department of Medicinal Plant and Mushroom Biotechnology, Faculty of Pharmacy, Jagiellonian University Medical College, 9 Medyczna St., 30-688 Krakow, Poland; monika.trepa@doctoral.uj.edu.pl; 2 SSG of Medicinal Plant and Mushroom Biotechnology, Faculty of Pharmacy, Jagiellonian University Medical College, 9 Medyczna St., 30-688 Krakow, Poland; dominika1.zych@student.uj.edu.pl

**Keywords:** *Ruta corsica*, endemic, in vitro cultures, furanocoumarins, furoquinoline alkaloids, HPLC analyses

## Abstract

*Ruta corsica* is a rare and endemic plant native to Corsica. Due to its limited distribution and the priority to preserve natural sites, has been insufficiently studied. In vitro cultures provide an opportunity to research *R. corsica* under controlled conditions. In the present study, in vitro cultures of *R. corsica* were conducted in Plantform^TM^ bioreactors. The study aimed to assess the effects of growth cycle length (5 and 6 weeks) and different concentrations of plant growth regulators (NAA and BAP) at 0.1/0.1, 0.1/0.5, 0.5/0.5, 0.5/1.0, and 1.0/1.0 mg/L on biomass growth and secondary metabolite accumulation. HPLC analysis identified compounds in the furanocoumarin and furoquinoline alkaloid groups, with furanocoumarins being the primary secondary metabolites (maximum total content: 1571.5 mg/100 g DW). Among them, xanthotoxin, psoralen, and bergapten were dominant, with maximum concentrations of 588.1, 426.6, and 325.2 mg/100 g DW, respectively. The maximum total content of furoquinoline alkaloids was 661 mg/100 g DW, with γ-fagarine as the primary metabolite, reaching 448 mg/100 g DW. The optimal conditions for secondary metabolite accumulation in *R. corsica* cultures were a 5-week growth cycle and the LS 0.1/0.1 medium variant.

## 1. Introduction

Plants have been integral to traditional medicine for centuries. In modern pharmacotherapy, they are used to obtain therapeutically valuable metabolites. Plant metabolites can constitute a medicinal raw material or can be used as substrates for the synthesis of derivatives that may have improved therapeutic values or lower toxicity [1]. Plants of the Ruta genus can also become a source of therapeutically valuable compounds [2]. However, collecting plant material from natural habitats poses significant challenges. Excessive harvesting can lead to habitat destruction, and many valuable medicinal plants are now protected species. Due to extinction risks, medicinal plant resources are predominantly obtained from cultivated fields, necessitating intensive fertilization and significant use of crop protection products like herbicides and fungicides. Climate change is also reducing cultivable land, prompting a search for alternative sources of medicinally valuable plant material. In vitro cultures have been intensively studied for this purpose for many years. Among these, highly organized cultures, such as shoot cultures, have shown the highest biosynthetic potential for producing biologically active metabolites [3]. The use of bioreactors in in vitro plant cultivation significantly enhances production efficiency, facilitating larger-scale, semi-industrial, or industrial production of plant materials [4,5]. *Ruta corsica* is an endemic species restricted to a limited geographic area, primarily found in Corsica’s mountainous regions above 450 m on loose, siliceous substrates like scree [6,7]. Its chemical composition is not well studied, with only limited reports on its different parts. Previous studies indicate that the *R. corsica* herb contains isoflavone-group flavonoids, including daidzein, genistein, prunetin, and formononetin [8], while its roots contain simple coumarins (corsicarin and isoscopoletin) and furanocoumarins (psoralen, xanthotoxin, and isopimpinellin), as well as furoquinoline alkaloids (skimmianine and dictamine) [9]. The essential oil from its aerial parts is rich in alkyl acetates, particularly nonyl acetate [10]. The chemical profile of *R. corsica* in vitro cultures has also been insufficiently explored. In prior studies, agitated *R. corsica* cultures demonstrated a high biosynthetic capacity for furanocoumarins and furoquinoline alkaloids. Comparative studies on agitated in vitro cultures of *R. chalepensis*, *R. corsica*, and *R. graveolens* revealed that all three accumulated these metabolites. Among them, *R. chalepensis* produced the highest amount of furanocoumarins, while *R. corsica* cultures were rich in furoquinoline alkaloids and exhibited the best antibacterial activity [11]. These findings prompted further investigation into scaling up *R. corsica* in vitro cultures using modern bioreactor systems, specifically the temporary immersion technique, which has proven effective for another rue species, *R. montana* [12].

In this study, we investigate, for the first time, the biosynthetic potential of *R. corsica* bioreactor cultures and optimize conditions for in vitro cultivation. Our research focuses on two dominant groups of secondary metabolites—linear furanocoumarins and furoquinoline alkaloids. Furanocoumarins, characterized by a benzo-α-pyrone core fused with a furan ring, exhibit various pharmacological activities, including anti-inflammatory, antibacterial, antifungal, antiviral, anticancer, anticonvulsant, and cytochrome P450 inhibitory effects [13,14]. Their photosensitizing properties, particularly of psoralen-type furanocoumarins like bergapten and xanthotoxin, under the influence of radiation with a wavelength of 320–400 nm (UVA light), combine with pyrimidine groups of DNA, which results in the temporary inhibition of cell proliferation. These compounds can be used in the treatment of psoriasis, acquired vitiligo, alopecia areata, eczema, and other skin diseases [15,16,17]. Furanocoumarins are used topically or orally in combination with UVA radiation. This is the so-called PUVA photochemotherapy method [18]. Another major group in *R. corsica* in vitro cultures are the furoquinoline alkaloids, known for antimicrobial and antiprotozoal activities, and with promising anti-inflammatory and AChE-inhibiting effects, which could be useful in neurodegenerative disease treatment [19,20].

## 2. Results and Discussion

### 2.1. In Vitro Bioreactor Cultures

In vitro cultures of *Ruta corsica* were conducted in Plantform^TM^ bioreactors for 5 and 6 weeks using five variants of LS medium with varying concentrations of plant growth regulators (PGRs). The auxin naphthyl-1-acetic acid (NAA) and cytokinin 6-benzylaminopurine (BAP) were applied at NAA/BAP ratios of 0.1/0.1, 0.1/0.5, 0.5/0.5, 0.5/1.0, and 1.0/1.0 mg/L, with each variant replicated three times.

**Figure 1:**
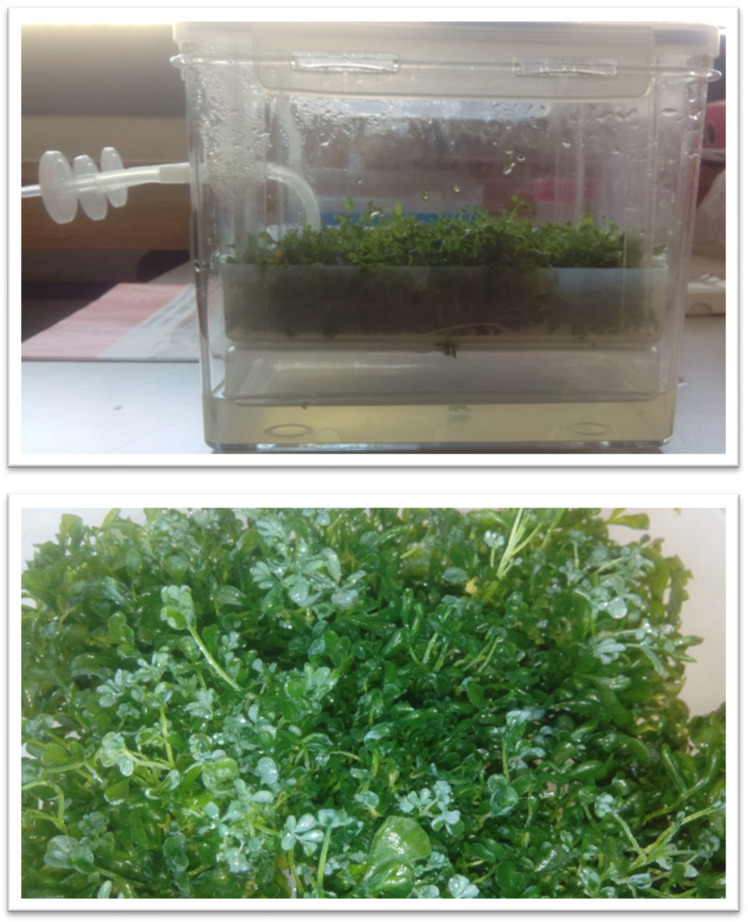
*R. corsica* cultures maintained in Plantform^TM^ bioreactor (LS 0.1/0.1 medium, 5-week growth cycle).

The cultures grew as green to dark green shoots with small leaves and partially developed root systems. Example bioreactor cultures are presented in Figure 1. The morphological appearance of the cultures did not change significantly depending on the LS medium variant used. The only differences in the morphology of the shoots concerned the stems. In the medium variants with a lower content of PGRs, the stems were longer and had a smaller diameter, whereas in the medium variants with higher concentrations of growth regulators, the stems were shorter but their diameter was larger.

**Figure 2:**
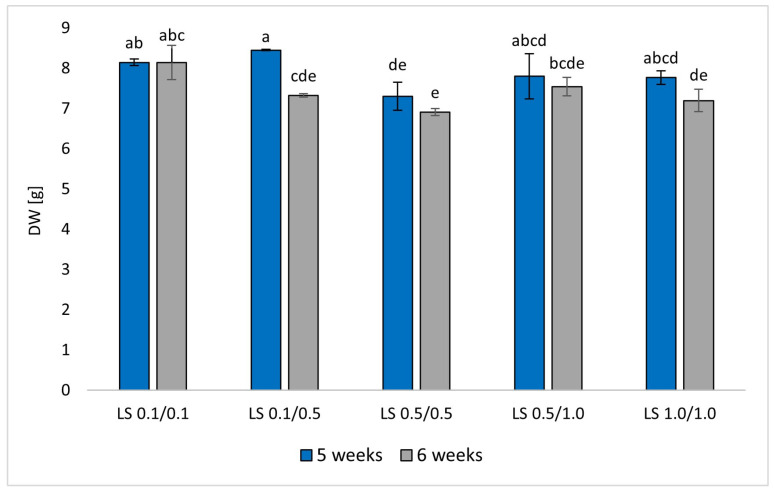
Results of dry weight DW [g] of R. corsica cultures grown in Plantform^TM^ bioreactors, two cultivation cycles (5 and 6 weeks), and 5 variants of LS medium with NAA/BAP ratio equal to 0.1/0.1, 0.1/0.5, 0.5/0.5, 0.5/1.0, and 1.0/1.0 mg/L, respectively. Means of three replicates ± SD. Letters a–e indicate significant differences at *p* < 0.05.

Dry weight (DW) measurements indicated minor variations between medium variants (Figure 2). All cultures showed significant biomass growth, approximately sevenfold, with slightly higher growth in media with lower PGR content. The highest DW (8.45 g) was recorded in LS 0.1/0.5, followed closely by LS 0.1/0.1 (8.15 g), both in 5-week cycles. The LS 0.5/0.5 variant, however, did not support notable biomass growth. The cultivation cycle had an impact on the amount of biomass obtained. In all medium variants, a 5-week cultivation cycle turned out to be more beneficial from an economic point of view. During the 6-week cultivation cycle, the inhibition of biomass growth and partially gradual shoot death were observed (Figure 2).

To date, there are no reports on biomass increase in in vitro cultures of *R. corsica* grown in bioreactors. Our previous studies on agitated cultures of this species in LS medium with NAA/BAP at 0.1/0.1 mg/L over 3, 4, 5, 6, and 7 weeks showed a significantly higher biomass increase. In a 5-week culture cycle, biomass increased 31.3-fold [11]. However, this type of culture is unsuitable for large-scale cultivation and mainly serves as experimental material for laboratory studies. Although biomass increase is lower in bioreactors, they allow for semi-industrial biomass production. Other in vitro cultures grown in bioreactors show similar biomass increase values. Biomass increase depends on the plant species and culture conditions (e.g., medium type and composition, and culture cycle length). For example, dry biomass increase in *R. montana* cultures in Plantform™ bioreactors ranged from 8.9-fold (LS 0.1/0.1, 6-week cycle) to 9.9-fold (LS 0.5/1.0, 5-week cycle). No significant differences were observed between LS medium variants or culture cycle length [12]. In this study, significant differences were found between cultures grown in 5-week and 6-week cycles. Further studies on *Schizandra chinensis* shoot cultures in Plantform^TM^ bioreactors using MS medium with 1.0/3.0 mg/L NAA/BAP showed biomass increases ranging from 2.5-fold after 30 days to 9-fold after 60 days [21]. Similarly, *Reynoutria japonica* shoot cultures showed a 6.5-fold biomass increase over a 5-week cycle (1/2 Murashige–Skoog MS medium, without PGRs). These bioreactor-grown shoots were also longer than those from other in vitro cultures [22]. *Scutellaria lateriflora* microshoot cultures kept in Plantform™ bioreactors on two medium types (LS and MS, both with 0.5/1.0 mg/L NAA/BAP) for 4 weeks showed a dry biomass increase of 6.3-fold on MS medium and 6.9-fold on LS medium [23].

### 2.2. Determination of Secondary Metabolite Content

**Figure 3:**
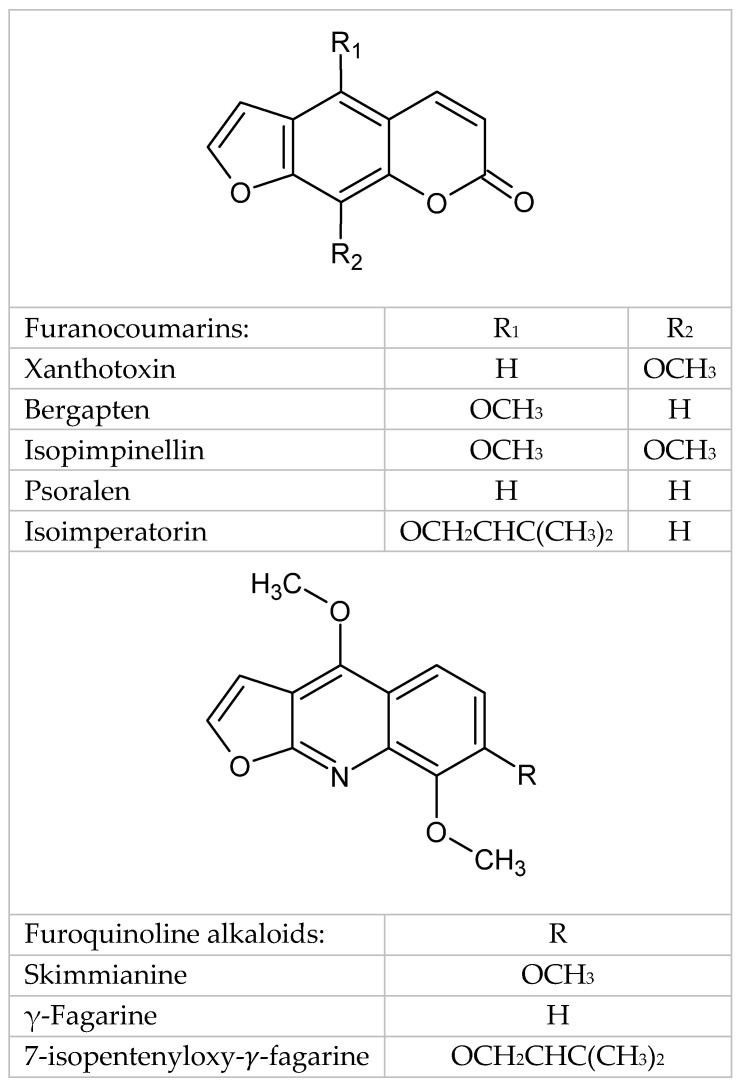
Chemical structures of analyzed secondary metabolites.

As a result of HPLC analyses, the presence of compounds from the group of linear furanocoumarins and furoquinoline alkaloids was found. Example chromatograms of the analyzed compounds and standard substances are provided in the Supplementary Materials (Figures S1 and S2). The chemical structures of the analyzed secondary metabolites are presented in Figure 3.

Furanocoumarins included xanthotoxin, psoralen, bergapten, isopimpinellin, and isoimperatorin. Xanthotoxin was the most abundant compound, with a maximum content of 588.1 mg/100 g DW, while psoralen and bergapten had maximum contents of 426.6 and 325.2 mg/100 g DW, respectively. The contents of isopimpinellin and isoimperatorin were lower at 160.8 and 70.8 mg/100 g DW, respectively. All maximum values were obtained in cultures maintained on the LS 0.1/0.1 medium over a 5-week cycle (Table 1).

For furoquinoline alkaloids, γ-fagarine, 7-isopentenyloxy-γ-fagarine, and skimmianine were detected. γ-fagarine had the highest content (448.3 mg/100 g DW), observed in the LS 0.1/0.1 medium after 5 weeks. Skimmianine was next, with a maximum content of 183.8 mg/100 g DW, while 7-isopentenyloxy-γ-fagarine had the lowest content at 29.3 mg/100 g DW. Like furanocoumarins, alkaloid maxima were found in the LS 0.1/0.1 medium over 5 weeks, whereas LS 0.5/1.0 and LS 1.0/1.0 were the least favorable for alkaloid accumulation (Table 2).

**Figure 4:**
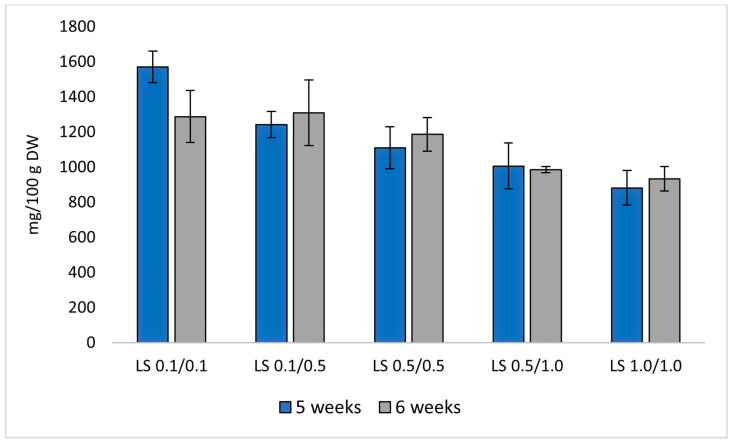
Total content of furanocoumarins [mg/g DW]. *R. corsica* cultures grown in Plantform^TM^ bioreactors, two cultivation cycles (5 and 6 weeks), and 5 variants of LS medium with NAA/BAP ratio equal to 0.1/0.1, 0.1/0.5, 0.5/0.5, 0.5/1.0, and 1.0/1.0 mg/L, respectively.

The highest total furanocoumarin content (1571.5 mg/100 g DW) was found in cultures grown on the LS medium variant containing 0.1/0.1 mg/L NAA/BAP (5-week culture cycle). The general trend shows that media containing a lower amount of PGRs are more conducive to accumulating this group of metabolites (Figure 4).

**Figure 5:**
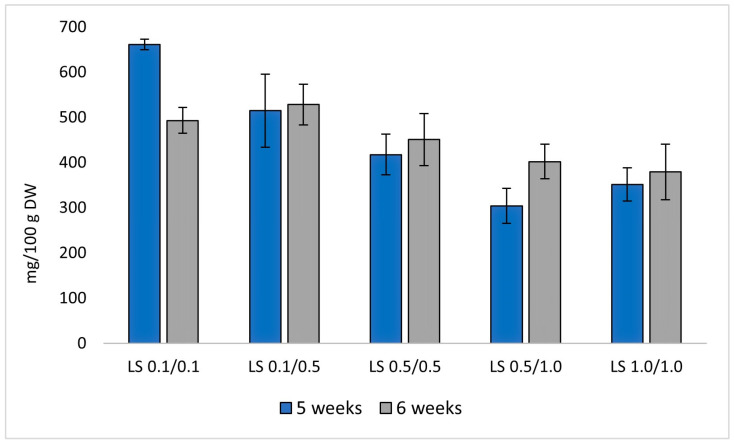
Total furoquinoline alkaloids [mg/100 g DW] obtained from *R. corsica* cultures grown in Plantform^TM^ bioreactors, two cultivation cycles (5 and 6 weeks) and 5 variants of LS medium with NAA/BAP ratio equal to 0.1/0.1, 0.1/0.5, 0.5/0.5, 0.5/1.0, and 1.0/1.0 mg/L, respectively.

For furoquinoline alkaloids, the highest total content (661.4 mg/100 g DW) was also found in the LS medium variant containing 0.1/0.1 mg/L NAA/BAP during 5 weeks, suggesting that lower PGRs enhance alkaloid accumulation (Figure 5).

Statistically significant differences were found only between media with the lowest and highest PGR content, with LS 1.0/1.0 being the least favorable for both furanocoumarins and furoquinoline alkaloids (Table 1 and Table 2).

In the case of the cultivation cycle, only for the LS 0.1/0.1 medium variant can it be clearly stated that a 5-week cultivation cycle is more beneficial for the production of the analyzed secondary metabolites. In the case of the other medium variants, there were no statistically significant differences in the content of individual compounds when using a 5-week and 6-week cultivation cycle (Figure 4 and Figure 5).

Modern bioreactors with a temporary immersion system (TIS), such as RITA^®^ and Plantform^TM^, are increasingly used for in vitro cultures, not only for micropropagation but also for secondary metabolite production. These systems allow for substantial biomass production within a short period [24] and are highly effective for cultivating shoot cultures on a larger scale, which has been technically challenging. In vitro shoot cultures are valuable for secondary metabolite production due to their organized tissue structure and high biosynthetic potential [3]. The use of bioreactors with a TIS for the production of secondary metabolites is very promising and therefore increasing number of researchers are dealing with this topic. This technique enabled the large-scale production of secondary metabolites from various groups of compounds. Studies on *Centella asiatica* shoot cultures conducted in Plantform^TM^ bioreactors have proven the very high potential of these cultures in the production of asiaticoside, phenolic acids, and flavonoids in *Centella asiatica* cultures [25], and therapeutically valuable lignans in in vitro cultures of *Schizandra chinensis* led the authors to the conclusion that the most advantageous for this purpose would be the use of the Plantform^TM^ bioreactor [21]. Promising results were also reported regarding the production of phenolic compounds in *Reynoutria japonica*, *Scutellaria lateriflora,* and *Salvia viridis* cultures grown in Plantform^TM^ bioreactors [22,23,26]. Cultures of the endemic plant *Salvia apiana* were studied as a source of essential oil. Cultures grown in bioreactors using the TIS proved to be very effective for obtaining the oil. The oil content was comparable in different bioreactors, and the differences were in the quantitative composition. The oil obtained from cultures grown in the RITA^®^ bioreactor contained ca. 42% cineole. In cultures from the Plantform^TM^ bioreactor, the content of cineole was higher (ca. 55%) [27].

To our knowledge, there are no publications in the literature on the qualitative and quantitative characteristics of the chemical composition of in vitro *R. corsica* cultures conducted in bioreactors. The only report on the subject of in vitro *R. corsica* cultures are the results of our previous studies on agitated cultures of this species. In the previous studies, extracts obtained from the biomass of *R. corsica* agitated cultures, conducted for a period of 3, 4, 5, 6, and 7 weeks on LS NAA/BAP 0.1/0.1 mg/L medium, were analyzed. The presence of compounds from the coumarin group, namely, xanthotoxin, bergapten, isoimperatorin, isopimpinellin, and psoralen, and from the alkaloid group, namely, γ-fagarine, 7-isopentenyloxy-γ-fagarine, and skimmianine, was detected. Among coumarins, the compound with the highest content was xanthotoxin (375.9 mg/100 g DW, the maximum content achieved in a 5-week culture), bergapten (174.7 mg/100 g DW, the maximum content achieved in a 7-week culture), and psoralen (278.7 mg/100 g DW, 6-week culture). Among alkaloids, γ-fagarine was the dominant compound, and its maximum content (133.4 mg/100 g DW) was achieved in a 4-week culture. The highest total content of coumarins (878.05 mg/100 g DW) was achieved in a 6-week culture, while in the case of alkaloids, the highest content (293.7 mg/100 g DW) was achieved in a 4-week culture [11]. Comparing the results obtained earlier with the current ones, it can be stated that bioreactor cultures are more beneficial for the production of metabolites from the above groups. The results obtained in this work are higher than in agitated cultures. Earlier research on agitated shoot cultures of *R. corsica* indicated that their metabolism is geared towards producing furanocoumarins and furoquinoline alkaloids. The findings of the current study also demonstrated a comparable capacity in cultures cultivated in bioreactors. The change in the metabolic profile is a phenomenon observed in in vitro cultures. It is probably due to the lack of expression of some genes encoding enzymes or the lack of some cofactors and the impairment of metabolic pathways [3].

The results of studies on other rue species also confirm the usefulness of TIS bioreactors for increasing the accumulation of secondary metabolites. *R. chalepensis* in vitro cultures were cultivated in RITA^®^ (VITROPIC, Saint-Mathieu-de-Treviers, France) bioreactors (4-week and 5-week growth cycles, with three variants of LS medium: 0.5/1.0, 0.1/0.1, and 1.0/1.0 NAA/BAP mg/L). The highest total content of furanocoumarins (1170 mg/100 g DW) was found in cultures grown on the LS medium variant containing 0.5/1.0 mg/L NAA/BAP (4-week growth cycle). The highest content of furoquinoline alkaloids (449 mg/100 g DW) was found in the LS medium variant containing 0.1/0.1 mg/L NAA/BAP (5-week growth cycle) [28]. Very high secondary metabolite production was obtained from the furanocoumarin group (with a maximum total content of 1824.3 mg/100 g DW) and the furoquinoline alkaloid group (with a maximum total content of 561.7 mg/100 g DW) in *R. montana* cultures grown in Plantform^TM^ bioreactors. The maximum contents of xanthotoxin (above 800 mg/100 g DW) and bergapten (above 400 mg/100 g DW) were extremely high [12]. The high level of these compounds was higher than in the parent plant. For comparison, the content of these compounds in field-cultivated plants was 410 and 110 mg/100 g DW, respectively [29]. Similarly to the present study, the highest content of the analyzed compounds was found in cultures grown on the LS 0.1/0.1 medium variant and a 5-week growth cycle.

The type and concentration of plant growth regulators (PGRs) used in in vitro cultures have a significant effect on cell differentiation, biomass growth, and the production of bioactive metabolites. The addition of PGRs to the media may increase the production of some metabolites, but on the other hand, it may also have a negative effect. In *Chonemorpha fragrance* cultures, higher concentrations of PGRs inhibited alkaloid production [3]. In *Ocimum americanum* L. var. *pilosum* cultures, the addition of 1 mg/L BAP to the medium caused a significant increase in rosmarinic acid accumulation compared to the control [30]. In turn, the use of the combination of NAA and BAP at a concentration of 2.5 mg/mL in MS medium had the best effect on the total content of phenolic compounds, flavonoids, and terpenoids in *Ocimum basilicum* cultures [31]. Depending on the type of PGRs used in in vitro cultures, an increase in the production of metabolites from specific groups is possible. In a *Lychnis flos-cuculi* callus culture, 2,4-Dichlorophenoxyacetic acid (2,4-D) showed an effect of increasing the production of triterpene saponins and ferulic acid derivatives. Dicamba promoted the accumulation of rutin, benzoic acid, and vitexin derivatives [32]. The content of salvinorin A in in vitro cultures of *Coleus scutellarioides* on a medium supplemented with NAA was three times higher than the value obtained on a medium without PGRs. On the other hand, in a medium containing BAP, the content of salvinorin A was three times lower than in control cultures [33].

The high metabolite contents obtained are a very promising result and a good starting point for further studies. In vitro cultures offer many strategies to increase the production of secondary metabolites. Among others, the addition of precursors of metabolic pathways or biotic and abiotic elicitation are possible. Such strategies allowed for the increased production of furanocoumarins and furoquinoline alkaloids in in vitro cultures of the most famous species of rue, *R. graveolens*. The earliest studies on *R. graveolens* cultures indicated that the use of biotic and abiotic stimulators could effectively improve furanocoumarin production. The impact of abiotic elicitors (benzothiazole and saccharin) was evaluated in agitated shoot cultures of *R. graveolens* using B5 medium during a 4-week growth cycle. The addition of 5% benzothiazole was found to increase furanocoumarin production, specifically, xanthotoxin (288.36 mg/100 g DW, an increase of 8.5 times compared to control cultures) and bergapten (153.78 mg/100 g DW, a 3.7-fold increase). The addition of benzothiazole increased the production of three alkaloids: γ-fagarine (5.8 mg/100 g DW, a 12-fold increase), kokusaginine (2.8 mg/100 g DW, a 5.3-fold increase), and skimmianine (6.4 mg/100 g DW, a 15.7-fold increase) [34]. Using another elicitor, chitin, also positively affected the furanocoumarin output, although to a slightly lesser extent: xanthotoxin (212 mg/100 g DW, a 6.3-fold increase) and bergapten (146 mg/100 g DW, a 3.5-fold increase). On the contrary, chitin was more efficient in enhancing the production of γ-fagarine (12.6 mg/100 g DW, a 36-fold increase), kokusaginine (4.4 mg/100 g DW, a 9-fold increase), and skimmianine (14.7 mg/100 g DW, a 25-fold increase). Furthermore, dictamnine (1.04 mg/100 g DW), not present in control samples, was produced [35]. The poorest outcomes came from using a bacterial lysate (*Bacillus* sp.) as an elicitor: xanthotoxin (153 mg/100 g DW, a 5-fold increase) and bergapten (90 mg/100 g DW, a 2-fold increase). Using a *Pectobacterium atrosepticum* bacterial lysate led to furoquinoline alkaloid contents as follows: γ-fagarine (68.0 mg/100 g DW), kokusaginine (17.2 mg/100 g DW), skimmianine (48.0 mg/100 g DW), and dictamnine (9.9 mg/100 g DW) [36].

## 3. Materials and Methods

### 3.1. In Vitro Cultures

In vitro cultures of *R. corsica* were established in 2016 in the Department of Pharmaceutical Botany, Faculty of Pharmacy, CM UJ, from seeds originating from the Botanical Garden of Maria Sklodowska-Curie University in Lublin.

The initial cultures for establishing the experiment in bioreactors were maintained in the form of liquid stationary cultures on LS medium according to [37], with the addition of plant growth and development regulators, i.e., NAA and BAP at a concentration of 1.0/1.0 mg/L.

Experimental cultures of *R. corsica* were conducted in Plantform^TM^ bioreactors, utilizing a temporary immersion system (Plant Form MWb & AJS, Hjärup, Sweden). In total, 15.0 g of previously cultivated biomass from in vitro cultures and 500 mL of liquid LS medium with the addition of growth and development regulators (auxin—NAA; cytokinin—BAP) in five NAA/BAP concentration variants (0.1/0.1, 0.1/0.5, 0.5/0.5, 0.5/1.0, and 1.0/1.0 mg/L) were placed in bioreactors. The biomass was flooded with the medium for 5 min every 90 min. The cultures were grown at a temperature of 24 ± 2 °C using constant artificial light (white Philips fluorescent lamps, 90 ± 2 μmol m^−2^ s^−1^). Five-week and six-week culture cycles were used, and then the obtained fresh mass was collected. The obtained biomass was filtered from the liquid medium, rinsed with distilled water, filtered again, and weighed. The next step was to dry the plant material at a temperature of approx. 38 °C. The dried biomass was weighed again and then micronized in a laboratory mortar.

### 3.2. Extraction

One gram of dry and powdered material from in vitro cultures was weighed into 250 mL round-bottom flasks. Extraction was carried out in 50 mL of methanol in a water bath for 2 h at the boiling temperature of the solvent, i.e., 64.7 °C. The prepared extracts were evaporated and the obtained dry residues were dissolved in 4.0 mL of HPLC-grade methanol, filtered through Millipore membrane filters with a pore size of 0.22 µm (Millipore, Bedford, MA, USA), and subjected to HPLC analysis in the next stage.

### 3.3. Reversed-Phase (RP)-HPLC Analysis

RP-HPLC analyses were conducted as previously described [38] on an HPLC apparatus (LaChrom Elite, Hitachi, Tokyo, Japan). DAD detection (DAD L-2455 detector, Hitachi, Tokyo, Japan) and a Purospher ^®^ RP-18e 250 × 4 mm/5 mm column (Merck, Darmstadt, Germany) were used. The mobile phase consisted of A—methanol; B—methanol/0.5% acetic acid 1:4 (*v*/*v*). The gradient elution was as follows: 100% B for 0–20 min; 100–80% B for 20–35 min; 80–60% B for 35–55 min; 60–0% B for 55–70 min; 0% B for 70–75 min; 0–100% B for 75–80 min; and 100% B for 80–90 min. The analysis was conducted at 25 °C, a flow rate of 1 mL min^−1^, and an injection volume of 10 µL, and the wavelength range was 200–400 nm. The quantification was performed at λ = 254 nm

The standards were purchased from the following companies: bergapten, xanthotoxin, and psoralen from Roth (Karlsruhe, Germany); skimmianine from ChromaDex (Irvine, CA, USA); and isoimperatorin, isopimpinellin, γ-fagarine, and 7-isopentenyloxy-γ-fagarine from ChemFaces (Wuhan, China). Methanol solutions of standard substances were used to prepare calibration curves. The following dilutions were used: 1, 0.5, 0.25, 0.125, and 0.0625 mg/mL. The calibration curve data for individual compounds are as follows: xanthotoxin (y = 100,000,000x + 3,000,000, R^2^ = 0.9984), bergapten (y = 100,000,000x + 559,286, R^2^ = 0.999), psoralen (y = 80,000,000x + 2,000,000, R^2^ = 0.9975), isoimperatorin (y = 100,000,000x + 2,000,000, R^2^ = 0.9994), isopimpinellin (y = 100,000,000x + 229,870, R^2^ = 0.9997), skimmianine (y = 100,000,000x + 3,000,000, R^2^ = 0.9976), γ-fagarine (y = 60,000,000x + 274,924, R^2^ = 0.9997), and 7-isopentenyloxy-γ-fagarine(y = 90,000,000x + 1,000,000, R^2^ = 0.999).

### 3.4. Statistical Analysis

A two-way analysis of variance (ANOVA), followed by Tukey’s post hoc test at *p* < 0.05, was performed using STATISTICA software v. 13.3 (StatSoft, Inc., Tulsa, OK, USA). The results were presented as ranges of three repetitions with standard deviation. A comparison of homogeneous groups (using the letters a–e, where “a” denotes the group with the highest mean, then “b” denotes the next mean, and so on down to the lowest means) was applied.

## 4. Conclusions

In this study, for the first time, the biosynthetic potential of *R. corsica* bioreactor cultures was examined and the conditions for the cultivation of in vitro cultures were optimized. Based on the obtained results, it can be concluded that *R. corsica* bioreactor cultures can be used as a biotechnological source of therapeutically valuable linear furanocoumarins (xanthotoxin and bergaptene) and furoquinoline alkaloids (γ-fagarine and skimmianine).

The use of modern bioreactors allows for obtaining large amounts of biomass, regardless of environmental conditions and season. Production can be carried out in a continuous system, becoming independent of climatic conditions. Bioreactor cultures can become a valuable alternative to traditional cultivation, especially during climate change. The next steps in research on *R. corsica* cultures will be the use of strategies to increase the production of secondary metabolites by changing the composition of media and lighting conditions, and adding precursors of metabolic pathways and elicitation.

## Figures and Tables

**Table 1:** Contents of furanocoumarins [mg/100 g DW] obtained from *R. corsica* cultures maintained in Plantform^TM^ bioreactors, depending on the duration of the growth cycle of the culture (5 and 6 weeks) and LS medium variant NAA/BAP mg/L (0.1/0.1, 0.1/0.5, 0.5/0.5, 0.5/1.0, and 1.0/1.0). Means of three repetitions ± SD. Different letters indicate significant differences (*p* < 0.05).

Compound	LS Medium Variant	Content [mg/100 g DW]	
	NAA/BAP mg/L	5-Week Growth Cycle	6-Week Growth Cycle
Xanthotoxin	0.1/0.1	588.10 ± 46.52 ^a^	558.31± 83.11 ^ab^
	0.1/0.5	471.48 ± 26.43 ^abcd^	527.27± 79.94 ^abc^
	0.5/0.5	402.35 ± 39.56 ^bcd^	418.41 ± 43.49 ^bcd^
	0.5/1.0	448.81 ± 62.05 ^bcd^	342.83 ± 17.48 ^d^
	1.0/1.0	352.81 ± 22.46 ^d^	355.52 ± 16.55 ^d^
Bergapten	0.1/0.1	325.18 ± 53.44 ^a^	253.20 ± 35.24 ^ab^
	0.1/0.5	241.74 ± 26.04 ^ab^	266.62 ± 11.28 ^ab^
	0.5/0.5	251.35 ± 21.59 ^ab^	270.57 ± 19.29 ^ab^
	0.5/1.0	214.25 ± 38.83 ^b^	262.53 ± 35.07 ^ab^
	1.0/1.0	189.84 ± 32.34 ^b^	245.40 ± 22.16 ^ab^
Isopimpinellin	0.1/0.1	160.82 ± 38.63 ^a^	90.05 ± 33.64 ^ab^
	0.1/0.5	121.07 ± 11.63 ^ab^	139.59 ± 35.70 ^ab^
	0.5/0.5	133.21 ± 27.49 ^ab^	140.35 ± 2.35 ^ab^
	0.5/1.0	92.20 ± 36.08 ^ab^	105.68 ± 28.18 ^ab^
	1.0/1.0	88.52 ± 24.92 ^ab^	68.23 ± 12.86 ^b^
Psoralen	0.1/0.1	426.64 ± 41.76 ^a^	326.70 ± 58.88 ^abc^
	0.1/0.5	345.55 ± 30.43 ^ab^	316.83 ± 75.54 ^abc^
	0.5/0.5	269.06 ± 38.33 ^bc^	296.17 ± 43.35 ^abc^
	0.5/1.0	203.05 ± 43.76 ^c^	222.34 ± 48.87 ^bc^
	1.0/1.0	206.02 ± 26.58 ^c^	208.98 ± 37.61 ^c^
Isoimperatorin	0.1/0.1	70.77 ± 1.77 ^a^	59.50 ± 3.42 ^bc^
	0.1/0.5	62.24 ± 3.08 ^ab^	59.81 ± 2.91 ^bc^
	0.5/0.5	54.89 ± 2.21 ^bcd^	60.99 ± 1.79 ^bc^
	0.5/1.0	48.34 ± 4.37 ^de^	52.66 ± 3.34 ^cde^
	1.0/1.0	45.11 ± 0.93 ^e^	55.58 ± 4.83 ^bcd^
Total furanocoumarins	0.1/0.1	1571.50 ± 89.37 ^a^	1287.75 ± 148.06 ^ab^
	0.1/0.5	1242.08 ± 75.57 ^bc^	1310.11 ± 187.36 ^ab^
	0.5/0.5	1110.86 ± 119.17 ^bcd^	1186.50 ± 95.76 ^bcd^
	0.5/1.0	1006.65 ± 130.10 ^bcd^	986.03 ± 16.47 ^bcd^
	1.0/1.0	882.30 ± 99.14 ^d^	933.71 ± 68.79 ^cd^

^abcde^ Letters denote homogenous groups.

**Table 2:** Contents of furoquinoline alkaloids [mg/100 g DW] obtained from *R. corsica* cultures maintained in Plantform^TM^ bioreactors, depending on the duration of the growth cycle of the culture (5 and 6 weeks) and LS medium variant NAA/BAP mg/L (0.1/0.1, 0.1/0.5, 0.5/0.5, 0.5/1.0, and 1.0/1.0). Means of three repetitions ± SD. Different letters indicate significant differences (*p* < 0.05).

Compound	LS Medium Variant	Content [mg/100 g DW]	
	NAA/BAP mg/L	5-Week Growth Cycle	6-Week Growth Cycle
Skimmianine	0.1/0.1	183.75 ± 17.62 ^a^	162.77 ± 20.14 ^abc^
	0.1/0.5	180.94 ± 35.67 ^a^	177.60 ± 35.83 ^ab^
	0.5/0.5	132.93 ± 5.25 ^abc^	142.77 ± 21.68 ^abc^
	0.5/1.0	91.60 ± 9.63 ^c^	138.20 ± 40.41 ^abc^
	1.0/1.0	98.73 ± 9.51 ^bc^	141.07 ± 47.07 ^abc^
γ-fagarine	0.1/0.1	448.31 ± 24.15 ^a^	296.95 ± 11.67 ^bcd^
	0.1/0.5	312.46 ± 44.84 ^bc^	330.57 ± 6.50 ^b^
	0.5/0.5	266.37 ± 46.59 ^bcde^	289.19 ± 36.09 ^abc^
	0.5/1.0	193.11 ± 31.90 ^e^	235.18 ± 15.29 ^cde^
	1.0/1.0	239.04 ± 33.87 ^cde^	219.78 ± 14.95 ^de^
7-isopentenyloxy-γ-fagarine	0.1/0.1	29.30 ± 2.13 ^a^	33.44 ± 4.37 ^a^
	0.1/0.5	21.40 ± 1.50 ^bc^	20.42 ± 2.60 ^cd^
	0.5/0.5	18.45 ± 1.87 ^cd^	19.08 ± 1.11 ^cd^
	0.5/1.0	19.22 ± 4.11 ^cd^	28.89 ± 1.92 ^ab^
	1.0/1.0	13.83 ± 1.04 ^d^	18.52 ± 2.93 ^cd^
Total furoquinoline alkaloids	0.1/0.1	661.36 ± 11.48 ^a^	493.17 ± 28.39 ^bc^
	0.1/0.5	514.80 ± 80.52 ^bc^	528.59 ± 44.91 ^ab^
	0.5/0.5	417.75 ± 45.22 ^bcde^	451.04 ± 57.84 ^bcd^
	0.5/1.0	303.93 ± 38.90 ^e^	402.27 ± 38.34 ^bcde^
	1.0/1.0	351.60 ± 36.81 ^de^	379.37 ± 61.39 ^cde^

^abcde^ Letters denote homogenous groups.

## Data Availability

Data are contained within the article and Supplementary Materials.

## Supplementary Materials

The following supporting information can be downloaded at: https://www.mdpi.com/article/10.3390/molecules29225261/s1, Figure S1: Sample chromatogram of the extract from *Ruta corsica* bioreactor cultures (LS 0.1/0.1 medium, 5-week growth cycle): 1. psoralen, 2. xanthotoxin, 3. isopimpinellin, 4. skimmianine, 5. bergapten, 6. γ-fagarine, 7. isoimperatorin, 8. 7-isopentenyloxy-γ-fagarine.; Figure S2: Sample chromatograms of the standard substances: 1. xanthotoxin, 2. psoralen, 3. bergapten, 4. isopimpinellin, 5. skimmianine, 6. isoimperatorin, 7. γ-fagarine, 8. 7-isopentenyloxy-γ-fagarine.
