# Supplementary material for: Optimization of the Production of Secondary Metabolites from Furanocoumarin and Furoquinoline Alkaloid Groups in In Vitro Ruta corsica Cultures Grown in Temporary Immersion Bioreactors
Source: Molecules. 2024 Nov 7;29(22):5261. doi: 10.3390/molecules29225261 (PMC11596115; doi:10.3390/molecules29225261)
Supplement: Supplementary file 1 [file molecules-29-05261-s001.zip › molecules-3263288-supplementary.pdf]

SUPPLEMENTARY MATERIALS

Optimization of the production of secondary metabolites from furanocoumarin and furoquinoline alkaloid groups in in vitro *Ruta corsica* cultures grown in temporary immersion bioreactors

Agnieszka Szewczyk, Monika Trepa, Dominika Zych

HPLC CHROMATOGRAMS

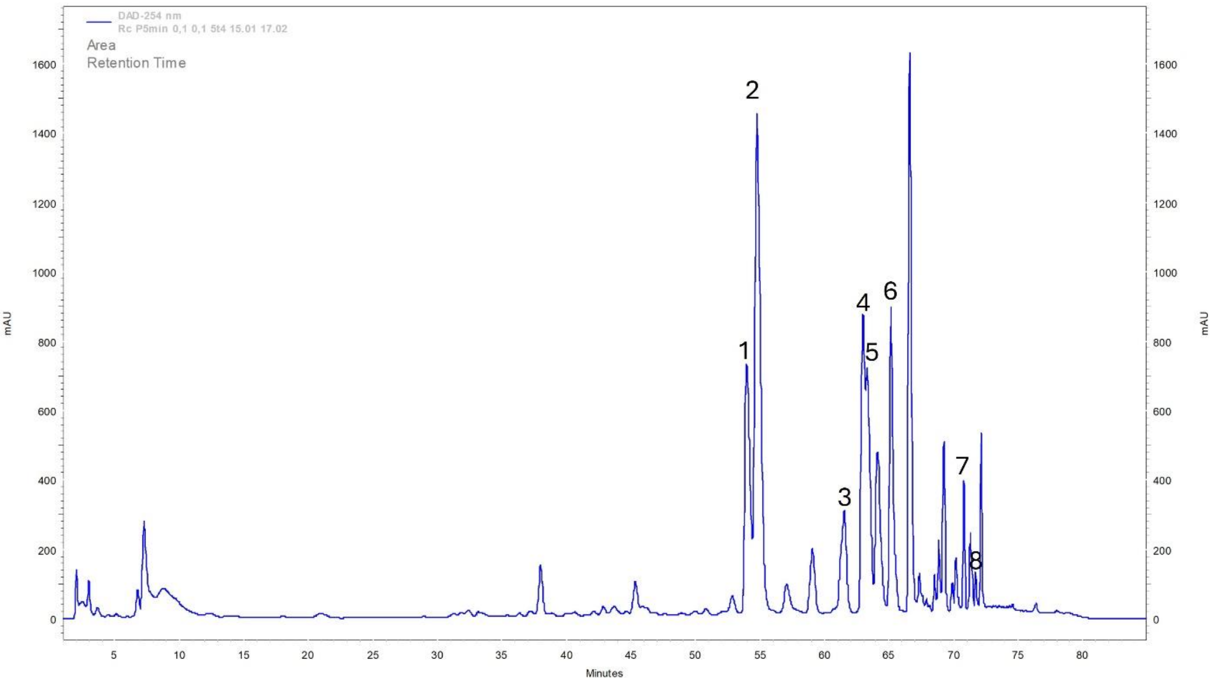

Figure S1 Sample chromatogram of the extract from *Ruta corsica* bioreactor cultures (LS 0.1/0.1 medium, 5-week growth cycle) 1. psoralen, 2. xanthotoxin, 3. isopimpinellin, 4. skimmianine, 5. bergapten, 6. γ-fagarine, 7. isoimperatorin, 8. 7-isopentenyloxy-γ-fagarine

xanthotoxin

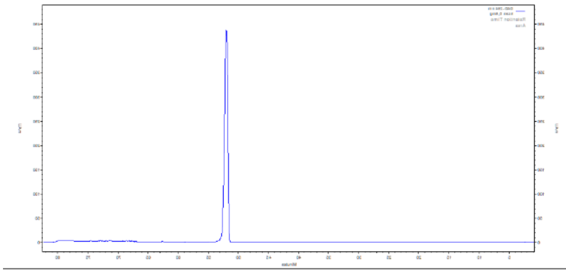

psoralen

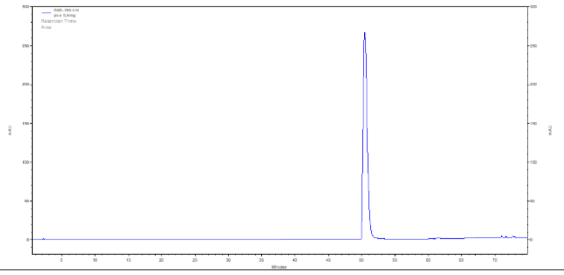

bergapten

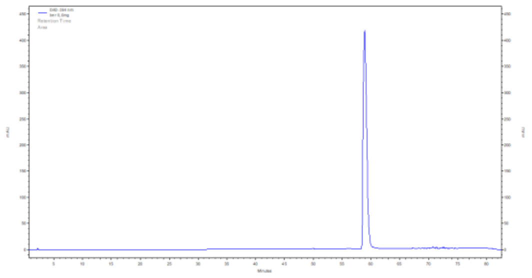

isopimpinellin

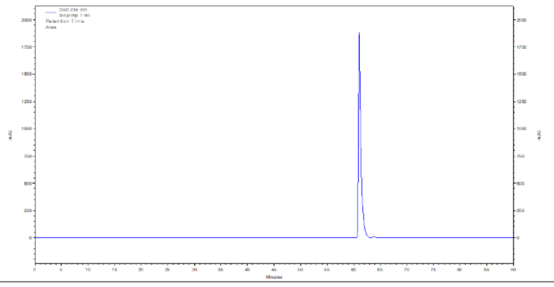

skimmianine

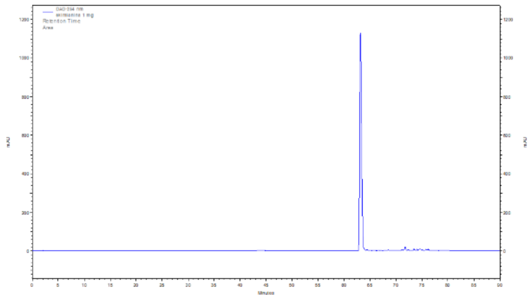

isoimperatorin

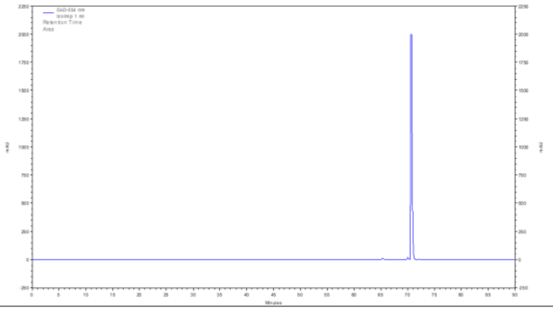

γ-fagarine

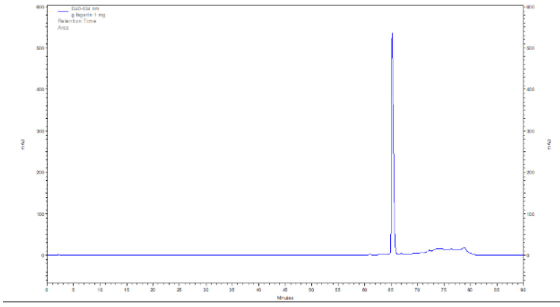

7-isopentenyloxy-γ-fagarine

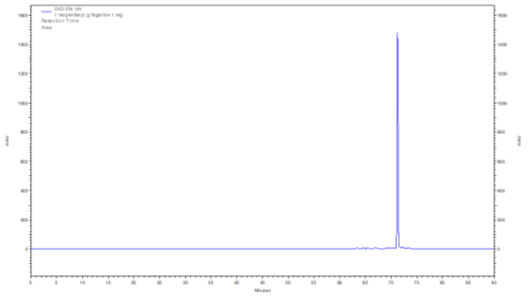

Figure S2 Sample chromatograms of the standard substances: 1. xanthotoxin, 2. psoralen, 3. bergapten, 4. isopimpinellin, 5. skimmianine, 6. isoimperatorin, 7. γ-fagarine, 8. 7-isopentenyloxy-γ-fagarine.
